# Screening, Identification, and Fermentation Characteristics of Lactic Acid Bacteria from Pickled Potherb Mustard and Potential Applications

**DOI:** 10.3390/foods14081431

**Published:** 2025-04-21

**Authors:** Xiaoxue Kong, Jiaxin Zhang, Hui Shen, Nan Shi, Hui Zhou, Yi Li, Yuxing Guo, Haibo Luo, Lijuan Yu

**Affiliations:** 1 Agro-Products Processing Research Institute, Yunnan Academy of Agricultural Sciences, Kunming 650221, China; kongxiaoxue@163.com (X.K.); shenhui@yaas.org.cn (H.S.); 2 School of Food Science and Pharmaceutical Engineering, Nanjing Normal University, Nanjing 210023, China; 45239@njnu.edu.cn (J.Z.); nnshinan@163.com (N.S.); huizhou0104@163.com (H.Z.); nnuliyi@163.com (Y.L.); 45222@njnu.edu.cn (Y.G.)

**Keywords:** starter, inoculation, microbial communities, high-throughput sequencing, antioxidant peptides

## Abstract

We identified strains of lactic acid bacteria from fermented potherb mustard that showed excellent fermentation properties. The goal was to identify superior starter cultures that would optimize the traditional fermentation process, reduce fermentation duration, and improve the quality of pickled potherb mustard. Four strains were screened: *Weissella cibaria* (LAB1, LAB3) and *Leuconostoc mesenteroides* (LAB2, LAB4). Then, after in vitro tests of tolerance to low pH and salt levels as well as lactic acid production ability, nitrite degradation ability, antibacterial properties, and antioxidant activity, LAB1 and LAB2 were selected as the best strains. Next, these two strains were used as starter cultures for fermenting potherb mustard. Each was inoculated into the fermentation solution. Compared to natural fermentation, both showed beneficial effects, including reducing nitrite content, shortening fermentation time, maintaining the reducing sugar, and increasing the levels of nitrogenous amino acids. Microbial diversity analyses revealed that, prior to fermentation, the predominant microbial communities were *Methylobacterium* and *Sphingomonas*, which primarily originated from the surrounding environment. However, 30 days after inoculation with the two strains, there was a significant increase in the abundance of *Weissella* and *Lactobacillus*, and *Weissella* emerged as the dominant bacterium. Inoculation of LAB1 effectively stabilized the bacterial community of the potherb mustard and significantly enhanced the content of nitrogenous amino acids in the final product, indicating that it is highly suitable as a mono-starter. On the other hand, LAB2 led to reduced nitrite content and facilitated the proliferation of *Weissella* and *Lactobacillus*, indicating that it is an effective mixed starter. Due to its limited effect on acid production, it is not recommended as a mono-starter for pickled mustard production.

## 1. Introduction

Potherb mustard (*Brassica juncea* Coss. Var. *multiceps* Tsen et Lee) is a biennial herb belonging to the *Brassicaceae* family, specifically, a variety of *Brassica juncea* Coss. It is commonly cultivated south of the Yangtze River basin in China [1]. The primary method of consuming potherb mustard is through pickling, which involves cleaning, dressing, and fermenting the raw material. It can be made at home or industrially and is very popular in Zhejiang Province. It has a processing and consumption history that spans several centuries and possesses a unique aroma and flavor; it is also abundant in various vitamins, minerals, and dietary fiber content [2]. It is a delicacy that has garnered significant appeal among both domestic and international consumers, thereby exhibiting immense market potential. In Ningbo alone, there are nearly 10 production facilities that have made noteworthy contributions to local economic development.

The traditional processing method of pickled potherb mustard (PPM) is so-called natural fermentation where the process is carried out by microorganisms adhering to the surface of raw mustard. Common microorganisms on mustard include lactic acid bacteria (LAB), yeasts, acetic acid bacteria, molds, and other undesirable microbes [3]. LAB play a crucial role in the fermentation process by producing lactic acid through metabolic activities, which lowers the pH value of the system and inhibits the growth of unwanted bacteria. They become dominant during fermentation and impart a distinctive flavor to pickles, making them an essential component of the process [4]. Zhang et al. [2] found that *Lactobacillus* was the principal bacteria in PPM under modified atmosphere conditions. Liu et al. [3] discovered that *Weissella* and *Lactobacillus* were the predominant bacterial genera in potherb mustard after natural fermentation for 30 d, and were related to changes in acidity, pH, and flavoring profiles (e.g., amino acids, organic acids, and alcohols).

However, traditional natural fermentation is subject to numerous uncontrollable factors including lengthy fermentation cycles, susceptibility to contamination by unwanted bacteria, and elevated nitrite levels, as well as inconsistent product quality and flavor across different batches produced in various factories [5,6]. Thus, to standardize the processing and production of PPM, it may be useful to screen and identify LAB with exceptional fermentation properties from high-quality, naturally fermented PPM, which could then be used as a starter culture [7].

Therefore, we isolated such LAB and comprehensively evaluated their fermentation characteristics and functional properties. Two identified strains were further used as direct starter cultures in potherb mustard; we compared and analyzed the resulting bacterial communities to establish an innovative fermentation procedure with predictable and good-quality results.

## 2. Materials and Methods

### 2.1. Material and Treatment

Pickled potherb mustard was supplied by Ningbo Yinzhou Sanfeng Kewei Food Co., Ltd., Ningbo, Zhejiang, China. *Staphylococcus aureus* CCTCC AB 2010020 and *Escherichia coli* CCTCC AB 2014080 strains were from China Center for Type Culture Collection (CCTCC). Fresh potherb mustard from Jiangsu Kangrun Agricultural Science and Technology Development Co., Ltd. (Nanjing, Jiangsu, China) was transported to our laboratory. Other ingredients including salt, sucrose, and white wine were purchased from local supermarkets in Nanjing, Jiangsu, China. The reagents used were of analytical purity and were purchased from Sinopharm Group Chemical Reagent Co., Ltd., Suzhou, Jiangsu, China.

### 2.2. Isolation of Bacterial Strains

Pickled potherb mustard samples weighing 5 g were aseptically transferred to a sterile homogenizing bag measuring 20 × 22 cm. Then, 45 mL sterile 0.85% normal saline (Land Bridge, Beijing, China) was added, and the mixture was homogenized at a rate of 8.0/s for 2 min using a homogenizer (HX-4, HuXi, Shanghai, China) to obtain a 1:10 sample homogenizing solution. This solution was further diluted 10-fold to achieve a final dilution ratio of 10^7^ times. Serial dilutions were plated on pre-solidified de Man, Rogosa Sharpe (MRS) agar (pH 6.2) (Land Bridge, Beijing, China) and incubated for 48 h at 37 °C under anaerobic conditions. Single colonies with distinct morphological characteristics were selected and numbered.

### 2.3. Molecular Identification

The Trelief^®^ Bacteria Genomic DNA Kit (TSP701–50) was used to extract genomic DNA. The concentration was assessed using a Qubit^®^ 4.0 fluorometer (ThermoFisher, Wilmington, DE, USA). Molecular identification of isolated strains was outsourced to Nanjing Qingke Biotechnology Co., Ltd. (Nanjing, China). Each sequence amplicon underwent BLAST^®^ analysis and alignment with the National Center for Biotechnology Information (NCBI) Sequence comparison database (https://www.ncbi.nlm.nih.gov/) to determine sequence identity and obtain a GenBank accession number. A phylogenetic tree was constructed using MEGA 6.0 software, incorporating sequences obtained from the NCBI Gene Bank.

### 2.4. Fermentation Characteristics of Isolated Bacteria

#### 2.4.1. Growth Curve and Acid Production Capacity

Activated LAB cultures were transferred to fresh MRS broth at a ratio of 2% and incubated at 37 °C for 28 h. For growth curve, samples were collected every 2 h and absorbances at 600 nm were measured (UV-1100, Mepida, Shanghai, China). Growth curves were plotted based on OD_600_ values. To assess acid production, the pH measurements were conducted every 2 h using a FE28 pH meter (Mettler Toledo Instruments (Shanghai) Co., Ltd., Shanghai, China). The titratable acidity (TA) was determined using the sodium hydroxide titration method outlined by Hou et al. [8], with uninoculated MRS broth used as a blank control. The results are expressed as grams of lactic acid per liter (g/L).

#### 2.4.2. Nitrite Degradation Capacity

The rate of degradation of sodium nitrite was determined using Hou’s method [8]. Activated LAB were inoculated into MRS broth of sodium nitrite at a 2% inoculation rate and incubated at 37 °C for 72 h. The initial concentration of sodium nitrite in the medium was set at 200 mg/L, while sterile distilled water served as a control group substitute. Absorbance values of the bacterial solution were measured at 540 nm for time intervals of 12 h, 24 h, 36 h, 48 h, and so on up to 72 h. The concentration of sodium nitrite was calculated based on a standard curve equation (y = 0.0287x + 0.0006, 0–12.5 μg). Finally, the degradation rate of sodium nitrite was calculated using the following formula:

The standard curve for sodium nitrite concentration is as follows:

$$\mathrm{Nitrite}\;\mathrm{degradation}\;\mathrm{rate}\;(\%)=\frac{D0-\mathrm{Dt}}{D0}\times 100\%$$

where D0 and Dt are the nitrite content (mg/L) in MRS broth before inoculation and after incubation, respectively.

#### 2.4.3. Acid and Salt Tolerances

The acid and salt tolerances of the LAB strains were performed as described by Oluwatosin et al. [9], with minor modifications. The bacterial solution was first activated at 37 °C for 24 h. To evaluate acid tolerance, the pH of the MRS broth was adjusted to 2.0, 3.0, 4.0, 5.0, 6.0, and 7.0 to create different acidic conditions. Then, the activated bacterial solution was inoculated into MRS broth at a rate of 2% and incubated at 37 °C for a duration of 24 h. Subsequently, the absorbance value at a wavelength of 600 nm was measured via spectrophotometry.

To assess the tolerance of the bacteria to sodium chloride (NaCl), the activated bacterial solution was inoculated into MRS broth with varying concentrations of NaCl (0%, 2%, 4%, 6%, 8%, and 10%) at an inoculation rate of 2%. Following incubation at 37 °C for an additional period of 24 h, the absorbance value at a wavelength of 600 nm was measured via spectrophotometry.

### 2.5. Assessment of Functional Properties

#### 2.5.1. Antibacterial Capacity

Antibacterial capacity was assessed using an agar well diffusion test [10]. *Staphylococcus aureus* CCTCC AB 2010020 and *Escherichia coli* CCTCC AB 2014080 preserved were utilized as indicator bacteria to detect antibacterial activity. Activated LAB solution was inoculated at a ratio of 2% and incubated for 18 h at 37 °C. Next, cell-free culture supernatants (CFSs) were prepared by centrifugation at 8000× *g* for 10 min. The pH of the CFSs were adjusted to approximately 7 by adding NaOH. Subsequently, suspensions of 10^7^ CFU/mL of each pathogen were prepared, and 100 μL was spread onto nutrient agar (NA). Four equidistant wells with a diameter of one centimeter were drilled into the NA plate using a punching machine. Three wells received 100 μL CFS, while the fourth received 0.85% sodium chloride solution as a control; all plates were cultured at 37 °C for 24 h before measuring the inhibition zone diameter in millimeters.

#### 2.5.2. Antioxidant Capacity

Cell-free culture supernatants were obtained as described in Section 2.5.1; pellets were washed three times in sterile phosphate-buffered saline (PBS, pH 7.2) and subsequently resuspended in PBS to obtain intact cells (CSs).

The scavenging activities of isolated LAB were assessed using a previously described method with some modifications [11]. A mixture of 1 mL CSs or CFSs and 0.2 mmol/L freshly prepared 2,2-diphenyl-1-picrylhydrazyl (DPPH) solution was briefly combined and allowed to react for 30 min in the absence of light. Blank samples contained deionized water. The reduction in absorbance at 517 nm was measured to monitor the scavenged DPPH. The scavenging ability was calculated as follows:

$$\mathrm{Scavenging}\;\mathrm{effect}\;(\%)=\frac{A_{517}\;(\mathrm{blank})\;-\;A_{517}\;(\mathrm{sample})}{A_{517}\;(\mathrm{blank})}\times 100\%$$

The ability to scavenge hydroxyl radicals was assessed using a previously described method with some modifications [12], namely, by quantifying the increase in absorbance at a wavelength of 536 nm. A blank containing an equal volume of distilled water was utilized as a substitute for CSs or CFSs, while a control group consisted of an equal volume of distilled water replacing hydrogen peroxide. Scavenging ability was calculated as follows:

$$\mathrm{Scavenging}\;\mathrm{effect}\;(\%)=\frac{A_{536}\;(\mathrm{sample})-A_{536}\;(\mathrm{blank})}{A_{536}\;(\mathrm{control})-A_{536}\;(\mathrm{blank})}\times 100\%$$

The scavenging of superoxide anion radicals was assessed using a previously described method with some modifications [13]. Blank samples were prepared using deionized water. Scavenged anions were quantified by measuring the decrease in absorbance at a wavelength of 325 nm. The scavenging ability was calculated as follows:

$$\mathrm{Scavenging}\;\mathrm{effect}\;(\%)=1-\frac{A_{325}\;\left(\mathrm{samples}\;\mathrm{and}\;\mathrm{pyrogallol}\right)-A_{325}\;(\mathrm{sample})}{A_{325}\;(\mathrm{pyrogallol})-A_{325}\;(\mathrm{blank})}\times 100\%$$

The reducing activity of LAB strains was determined following the method of Vaez et al. [14] The absorbance at a wavelength of 700 nm was measured to determine the level of reducing activity, where more absorbance indicated a greater degree of reducing activity. Distilled water served as a blank control; it was mixed with potassium ferricyanide and PBS. The calculation was as follows:

$$\mathrm{Reducing}\;\mathrm{activity}\;(\%)=\frac{A_{700}\;(\mathrm{sample})\;-A_{700}\;(\mathrm{blank})}{A_{700}\;(\mathrm{blank})}\times 100\%$$

#### 2.5.3. Identification of Peptides and Antioxidant Capacity

The activated strain LAB1 was inoculated into MRS broth and incubated at 37 °C for 48 h. The polypeptides in CFSs were identified using liquid chromatography–mass spectrometry (LC-MS/MS) analysis conducted by Beijing Baitai Parker Biotechnology Co., Ltd. (Beijing, China), with uninoculated MRS broth used as a control. The online Peptide Ranker Score (http://distilldeep.ucd.ie/PeptideRanker/, accessed on 8 July 2023) was utilized to predict peptide bioactivity with scores greater than 0.5 selected through screening processes [15]. Select peptides were chemically synthesized by Nanjing Synpeptide Co., Ltd. (Nanjing, China).

To assess the DPPH radical-scavenging capacity, 0.5 mL synthetic peptides (5mg/mL) were mixed with 0.5 mL DPPH radical working solution (0.1 mmol/L) in ethanol. The absorbance at 517 nm was measured, and the scavenging rate was calculated as in Section 2.5.2. The same concentrations of ascorbic acid and glutathione were used as control.

The 2,2′-azinobis(3-ethylbenzothiazoline-6-sulfonic acid) (ABTS^+^) radical scavenging activity was assessed following the method of Vaez et al. [14], with some modifications. ABTS^+^ was generated by freshly reacting ABTS+ (7 mM) with K_2_S_2_O_8_ (2.45 mM). The mixture was left at room temperature in the dark for 12 h, and diluted with ethanol to an absorbance of 0.70 ± 0.20. After mixing 0.5 mL ABTS^+^ solution and 0.5 mL synthetic peptides (5 mg/mL), the solution was incubated at room temperature for 6 min. Absorbance was measured at 734 nm. The scavenging rate was calculated as follows:

$$\mathrm{Scavenging}\;\mathrm{rate}\;\left(\%\right)=\frac{A_{734}\;(\mathrm{blank})-A_{734}\;(\mathrm{sample})\;}{A_{734}\;(\mathrm{blank})}\times 100$$

### 2.6. Inoculation Fermentation of Potherb Mustard (Brassica juncea var. crispifolia)

In fermentation tests, the culture media of different strains of bacteria were incubated for 24 h at 37 °C, followed by centrifugation at 8000× *g* for 10 min at 4 °C. The supernatants were discarded and the collected bacteria were washed three times with 0.85% sodium chloride solution before being suspended in normal saline (1.0 × 10^10^ CFU/mL) for inoculation.

**Figure A1:**
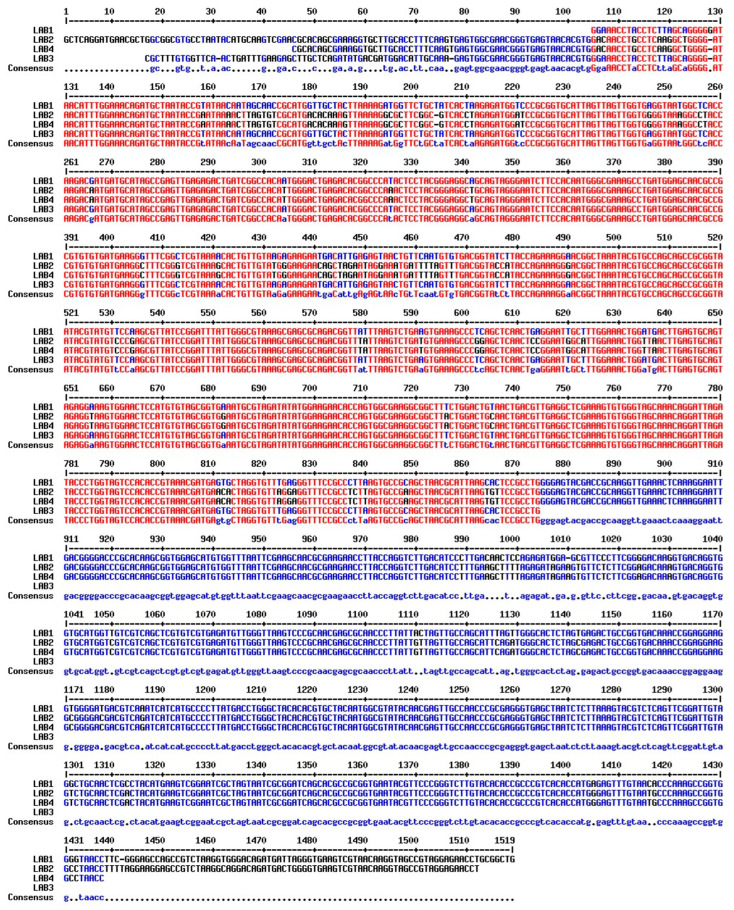
Multiple alignments of the lactic acid bacteria genes isolated from pickled potherb mustard.

**Figure A2:**
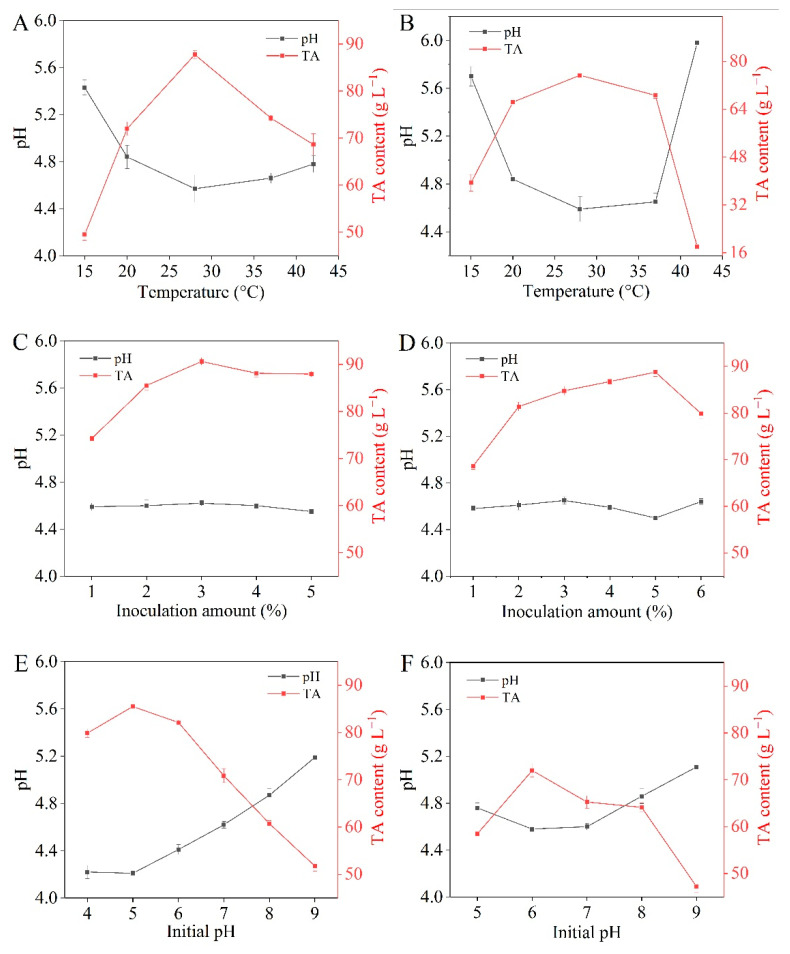
Effect of temperature (**A**,**B**), inoculation amount (**C**,**D**), and initial pH (**E**,**F**) on pH and total acid content in LAB1 and LAB2 fermented liquid culture medium.

**Figure A3:**
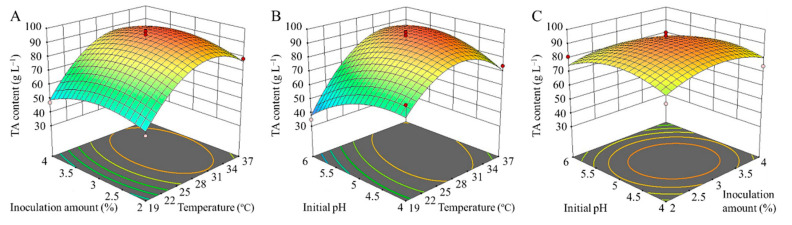

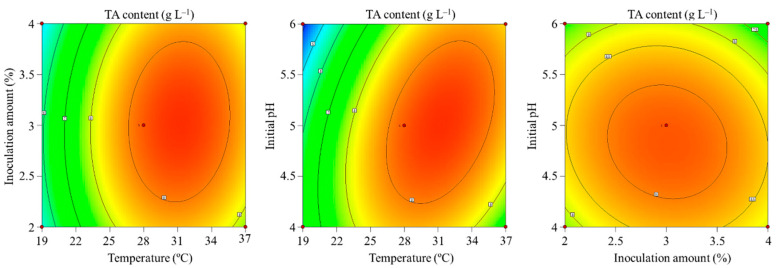
(**A**) Inoculation amount (%) and Temperature (°C); (**B**) Initial pH and Temperature (°C); (**C**) Initial pH and Inoculation amount (%). Response surface map and contour map of the interaction of various factors on the total acid content (LAB1).

The fresh potherb mustard (60 kg) was placed in a stainless steel container (80 L) and thoroughly mixed with 60 g/kg salt, and then a stainless steel cover and heavy object, such as a stone, were placed on the top of the vegetables (*w*:*w* = 1:1). The vegetables were pickled at ambient temperature for 1 d until the potherb mustard was immersed in the juice caused by the dehydration of plant cells. After pickling, the vegetables were randomly divided into four equal groups (15 kg each). Three groups were transferred to glass jars and inoculated with 1.0 × 10^10^ CFU/mL, 3% (*v*:*w*) LAB1, LAB2, and LAB1 + LAB2 (LAB1:LAB2 = 2:1), respectively. One group without inoculation of LAB was prepared as the control. Then, jar lids were tightly closed and the samples were fermented at ambient temperature for 30 d. Three biological replicates were established in each group. The optimal fermentation conditions were determined using a response surface experiment (Table A1, Table A2, Table A3, Table A4, Table A5 and Table A6 and Figure A1, Figure A2 and Figure A3 in Appendix A).

### 2.7. Chemical Analysis of Fermented Potherb Mustard

The pH and total acidity (TA) were analyzed after homogenizing 5 g fermented mustard with 20 mL deionized water. The results are expressed as grams of lactic acid per kilogram fresh weight (g/kg). Nitrite levels were determined by adding 5 g fermented mustard to 20 mL deionized water to create a homogenate. The results are expressed as mg/kg. The content of reducing sugar (RS) was determined using Zhou’s method [4], and the results are expressed as g/kg. Nitrogenous amino acids (NAAs) were measured following Wu’s method [16], and the results are expressed as g/kg.

### 2.8. Microbial Diversity Analyses

The samples were divided into five groups: natural fermentation for 0 and 31 d (controls), LAB1 fermentation for 31 d, LAB2 fermentation for 31 d, and mixed fermentation of both LAB1 and LAB2 at a ratio of 2:1 for 31 d. These samples were sent to Shenggong Bioengineering Co., Ltd. (Shanghai, China) for Illumina Miseq high-throughput sequencing and microbial diversity analysis.

The obtained double-ended sequence data were processed by removing primers and junction sequences. Samples were then identified and distinguished based on the barcode label sequence to obtain individual sample data. Finally, a quality control step was performed to filter out any invalid data. The non-repetitive sequences (excluding single sequences) were clustered into operational taxonomic units (OTUs) using a 97% similarity threshold. Feature-level alpha diversity indices, including the observed richness (Sobs), the Chao1 estimator, and the ACE estimator, were employed to quantify community richness. The Shannon index, Simpson index, and Good’s coverage were utilized to evaluate community diversity, while the Shannoneven index was used to assess community evenness.

### 2.9. Statistical Analyses

The data are presented as means ± standard deviation (*n* = 3). Statistical analyses were performed using SPSS software (version 26.0, SPSS Inc., Chicago, IL, USA). Student’s *t*-tests and one-way ANOVA followed by Duncan’s multiple-range test were employed to determine statistical differences between different fermentation stages; *p*-values < 0.05 were taken to indicate significant differences.

## 3. Results

### 3.1. Isolation of LAB

**Figure 1:**
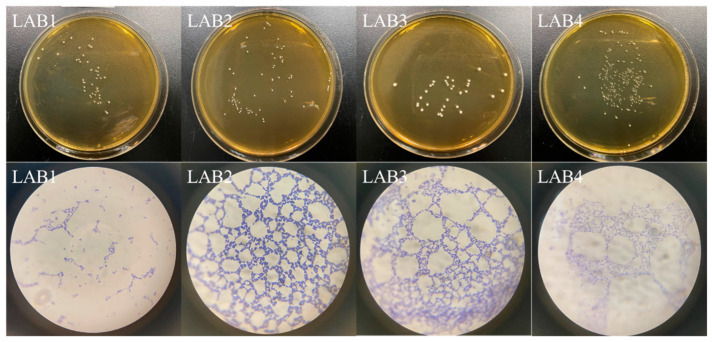
Colony and thallus morphology of four lactic acid bacteria strains isolated from the pickled potherb mustard.

We obtained four pure bacterial colonies, namely, LAB1, LAB2, LAB3, and LAB4. These isolates were preserved in the probiotic library maintained by the Department of Food Science at Nanjing Normal University. Following purification, colonies of each strain were added to MRS plates, where they eventually exhibited a white or milky-white coloration; they were round with smooth surfaces and well-defined edges (Table A7). They also displayed positive Gram staining characteristics. Under a microscope, the bacteria appeared as rod-shaped cells arranged individually, in pairs, or in chains (Figure 1).

### 3.2. Molecular Identification

**Figure 2:**
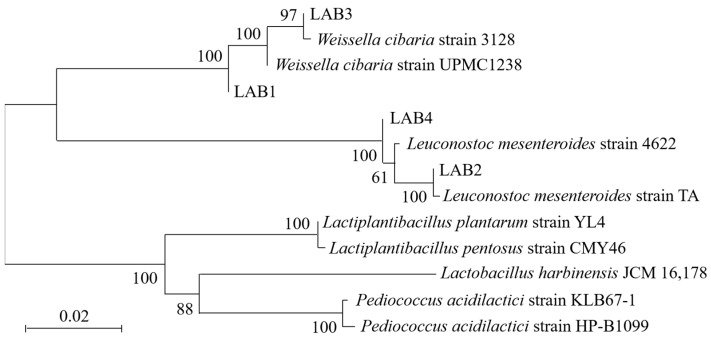
The phylogenetic trees of four lactic acid bacteria strains isolated from the pickled potherb mustard.

**Figure A4:**
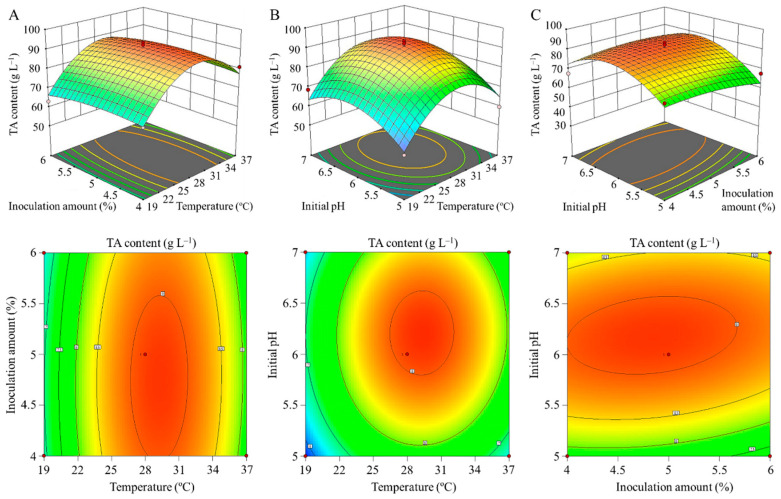
(**A**) Inoculation amount (%) and Temperature (°C); (**B**) Initial pH and Temperature (°C); (**C**) Initial pH and Inoculation amount (%). Response surface map and contour map of the interaction of various factors on the total acid content (LAB2).

The bacteria were subsequently identified through biochemical and physiological characteristics (Table A8) and 16S rRNA sequencing (Appendix B). The NCBI database was utilized to conduct similarity and sequence homology comparisons (Figure A4), while a phylogenetic tree was constructed using MEGA 11.0 software (Figure 2). The identified species were *Weissella cibaria* (LAB1, LAB3) and *Leuconostoc mesenteroides* (LAB2, LAB4) (Table 1).

### 3.3. Fermentation Characteristics of LAB

#### 3.3.1. Growth Curve and Acid Production Capacity

**Figure 3:**
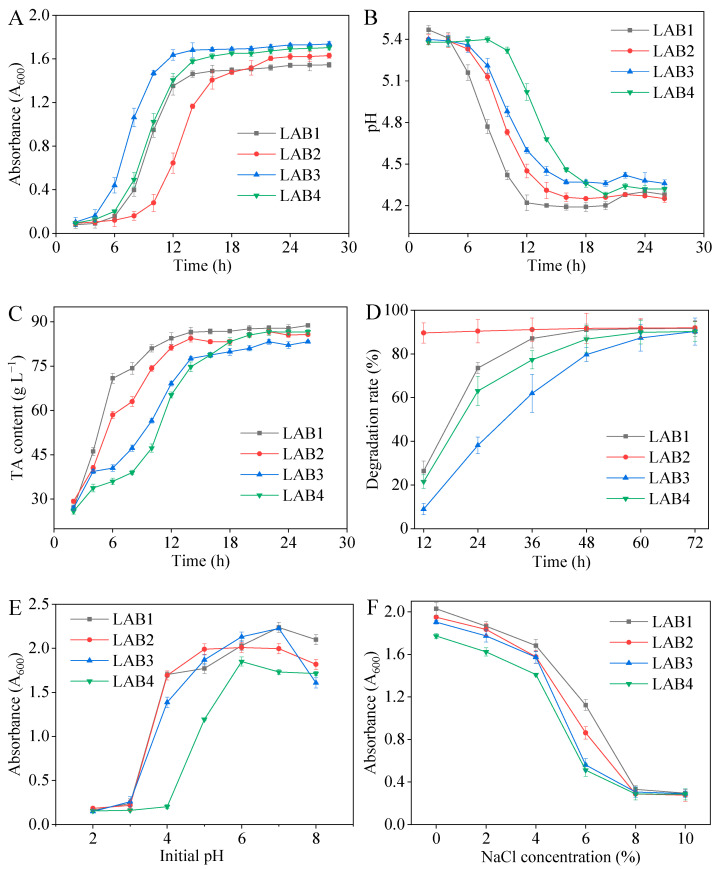
Growth curve (**A**), pH value (**B**), titratable acidity (**C**), nitrite degrade ability (**D**), and acid (**E**) and salt tolerance (**F**) of four lactic acid bacteria strains isolated from the pickled potherb mustard.

The ideal starter should possess vigorous growth ability and robust acidogenic capability, enabling it to rapidly decrease the pH level of the fermentation environment during the initial stages. The growth curves of the four strains are depicted in Figure 3A. LAB3 exhibited the most rapid growth rate, entering the logarithmic phase within 6 h, followed by LAB1 and LAB4 at approximately 8 h, and finally LAB2 at around 12 h. The acid-producing capacity of LAB1 was highest, effectively reducing the environmental pH below 4.5 within a mere 12 h of fermentation. The TA was equivalent to 84.4 g/L (Figure 3B,C).

#### 3.3.2. Nitrite Degradation Activity

Nitrite degradation was observed within a time frame of 12 to 72 h. All four strains degraded nitrite, with LAB2 showing the highest proficiency (*p* < 0.05). Within a span of 12 h, LAB2 achieved a remarkable degradation rate exceeding 85% (Figure 3D and Table A9). In addition, LAB3 exhibited the lowest nitrite degradation rate, potentially due to slower adaptation compared to LAB1 and LAB2.

#### 3.3.3. Acid and Salt Tolerances

A high-quality starter should be able to withstand a pH of approximately 4.0, thereby enhancing fermentation efficiency and shortening fermentation time. The growth of all four strains was inhibited at pH levels of 2.0 and 3.0. All but LAB4 showed robust growth at a pH of 4.0. LAB4 was optimal at pH 6.0 and the remaining three were optimal at pH 7.0 (Figure 3E). The higher lag phase of LAB4 suggests possible metabolic adaptations that require further exploration.

The NaCl content of pickled potherb mustard generally exceeds 5%, and the salt tolerance of the four strains is presented in Figure 3F. Under no-salt conditions, all four strains exhibited optimal growth, while their growth was significantly (*p* < 0.05) inhibited by salt concentrations exceeding 8%. In a culture with 6% salt concentration, LAB1 and LAB2 demonstrated significantly better growth than LAB3 and LAB4, with LAB1 exhibiting the highest level of salt tolerance (*p* < 0.05).

### 3.4. Functional Properties of LAB

#### 3.4.1. Antibacterial Capacity

**Figure A5:**
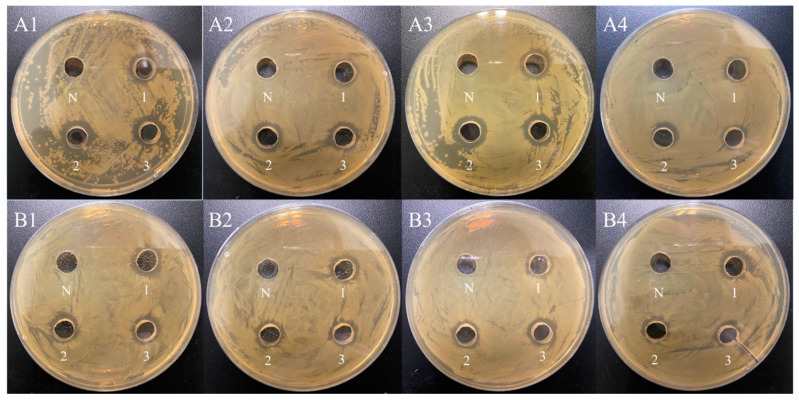
Inhibition of lactic acid bacteria on *Escherichia coli* (**A**) and *Staphylococcus aureus* (**B**). (**A1**–**A4**) and (**B1**–**B4**), LAB1-LAB4; N, control; 1, 2, 3, three replicates.

To assess the antibacterial efficacy of the four strains, *S. aureus* CCTCC AB 2010020 and *E. coli* CCTCC AB 2014080 were inoculated onto plates and tested with isolated LAB CFSs. The experimental results are presented in Table 2 and Figure A5. All four strains exhibited inhibitory effects against both *S. aureus* and *E. coli*, with LAB1 and LAB3 of the same *Weissella* genus displaying superior inhibitory effects against *E. coli*, particularly LAB1 (*p* < 0.05).

#### 3.4.2. Antioxidant Capacity

**Figure 4:**
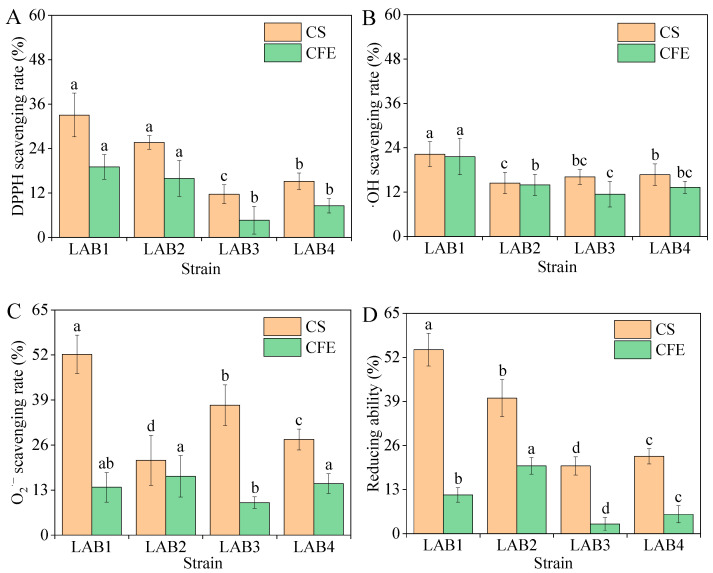
DPPH (**A**), OH (**B**), and O_2_^−^ (**C**) scavenging rate and reducing ability (**D**) of four lactic acid bacteria strains isolated from the pickled potherb mustard. Within the same index, different lowercase letters are significantly different (*p* < 0.05). CS, cell suspension; CFE, cell-free extract; DPPH, 2,2-diphenyl-1-picrylhydrazyl; ·OH, hydroxyl radical; O_2_^·−^, superoxide anion.

We assessed the antioxidant capacity of CSs and CFSs derived from the four isolated LAB strains; the results are presented in Figure 4 and Table A10. The scavenging rates of DPPH, hydroxyl radicals, and superoxide anions, as well as the reducing activity of CSs, were all stronger compared to those of CFSs. Among the four strains evaluated, LAB1 showed the most potent antioxidant capacity (*p* < 0.05). Therefore, we evaluated the ability of LAB1 to produce antioxidant peptides in MRS broth.

#### 3.4.3. Evaluation of Antioxidant Peptides

**Figure 5:**
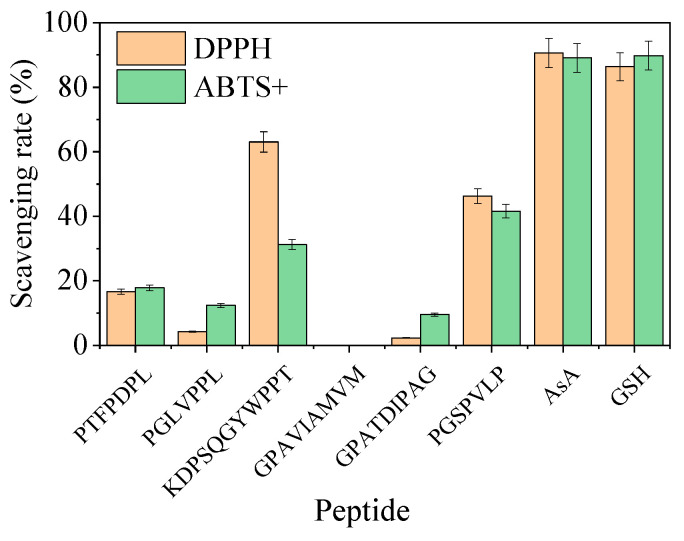
DPPH and ABTS+ scavenging rate of six synthetic peptides. DPPH, 2,2-diphenyl-1-picrylhydrazyl; ABTS+, 2,2′-azinobis (3-ethylbenzothiazoline-6-sulfonic acid); AsA, ascorbic acid; GSH, glutathione.

The peptide scores of 12 peptides exceeded 0.5, encompassing amino acid residues ranging from 7 to 15. The six peptide chains with the highest scores were chemically synthesized and their in vitro antioxidant capabilities were evaluated based on the clearance rates of DPPH and ABTS^+^ (Table 3). Although the DPPH and ABTS^+^ scavenging rates of all synthetic peptides were lower compared to those of ascorbic acid and glutathione at equivalent concentrations, the synthetic peptide KDPSQGYWPPT exhibited a relatively high scavenging rate on DPPH at 63.0%, while PGSPVLP showed a peak scavenging rate on ABTS^+^ at 41.6% (Figure 5). These results indicated that the LAB1 inoculation could increase the antioxidant activity of MRS broth, which may be associated with the generation of antioxidant peptides.

### 3.5. Fermentation of Mustard

**Figure 6:**
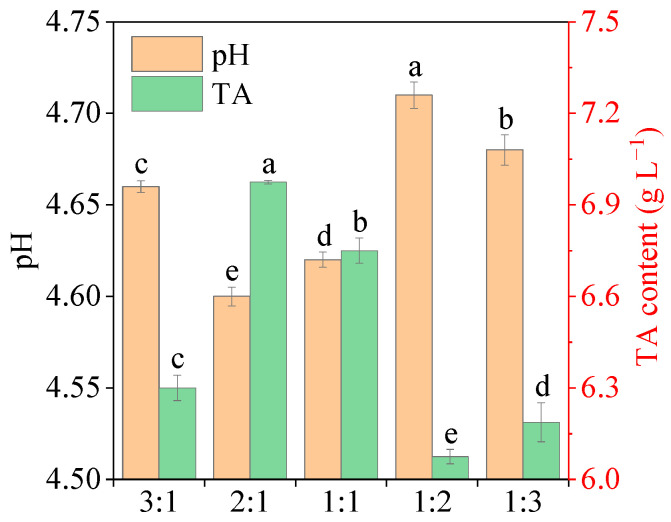
Effect of different proportions of lactic acid bacteria (LAB1:LAB2) inoculation on pH and total acid of MRS broth after incubation for 24 h at 37 °C. Within the same index, different lowercase letters are significantly different (*p* < 0.05).

The impact of varying proportions of LAB (LAB1:LAB2) on pH and TA is illustrated in Figure 6. At a ratio of 2:1, the fermentation system achieved the lowest pH value and highest TA (*p* < 0.05). Consequently, the optimal inoculation ratio for mixed fermentation was determined to be 2:1 (LAB1/LAB2).

**Figure 7:**
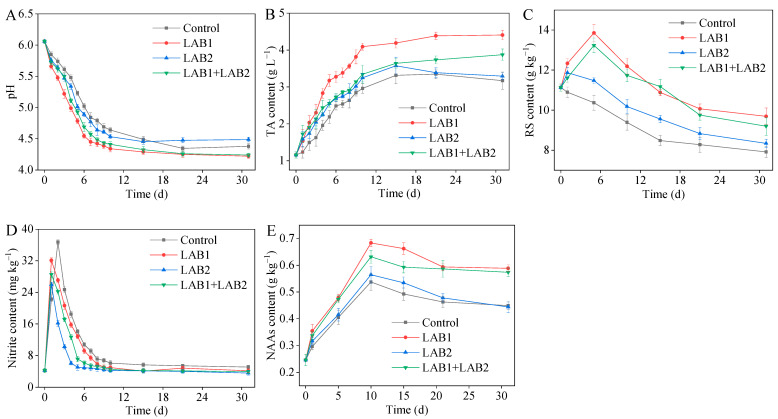
pH (**A**), TA (**B**), RS (**C**), nitrite (**D**), and NAA (**E**) contents in the potherb mustard during fermentation. TA, titratable acidity; RS, reducing sugar; NAA, nitrogenous amino acid.

The changes in pH and TA during fermentation are presented in Figure 7A,B. The initial pH was 6.06 and the TA was 1.2 g/L on day 0. After a fermentation period of 10 days, both LAB1 and LAB1+LAB2 exhibited pH values below 4.5, with LAB1 reaching a minimum pH of 4.34 and TA exceeding 4.0 g/L. It is worth noting that inoculation with LAB1 significantly accelerated the decline in pH during fermentation, thereby facilitating its overall progress.

The RS in potherb mustard serves as a source of energy for microbial growth during fermentation. The changes in RS are presented in Figure 7C. Throughout the fermentation process, the control group consistently exhibited lower levels of RS compared to all three inoculation groups. Specifically, LAB1 and LAB1+LAB2 showed an initial increase in RS until 5 days before fermentation, followed by a gradual decline thereafter. By day 31, when fermentation concluded, the LAB1 group had significantly higher levels of RS than any other group (*p* < 0.05).

Nitrite is a crucial indicator of food safety. On day 0, nitrite levels were 4.24 mg/kg in all four groups. However, 1 day after inoculation fermentation, the levels peaked at 32.11 mg/kg, 25.97 mg/kg, and 28.55 mg/kg in LAB1, LAB2, and LAB1+LAB2, respectively. In contrast, on the second day of natural fermentation it peaked at 36.73 mg/kg (control group). Following these peak values, there was a rapid decrease across all samples, with LAB2 exhibiting the fastest decline to reach a level of 6 mg/kg by day 4; this was followed by the mixed fermentation group (LAB1+LAB2), which reached this level by day 6; LAB1, which achieved it by day 8; and the controls, which attained it by day 10. After this initial rapid decline, all groups experienced a gradual reduction in nitrite content until day 31; at this point, all groups except controls had lower levels compared to before fermentation (Figure 7D).

The nitrogen content of amino acids (AAN) can serve as an indicator of the flavor profile of potherb mustard to some extent, with its levels being directly proportional to the complexity of the final product’s taste. We found that during the initial 10 days of fermentation, all four groups experienced rapid growth in NAAs, followed by a decline from day 10 to day 20 and eventually stabilizing after day 20. At the end of fermentation, both LAB1 and LAB1+LAB2 exhibited significantly (*p* < 0.05) higher levels of NAAs than the other two groups, indicating more complex flavor and overall quality (Figure 7E).

### 3.6. Microbial Community Composition

**Figure A6:**
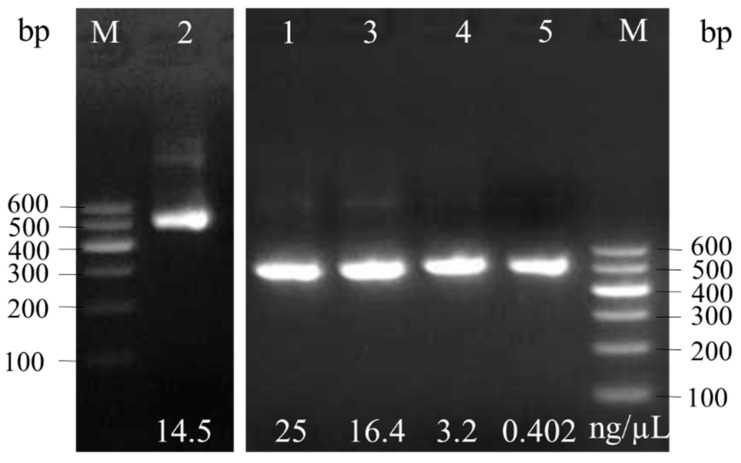
Electrophoretogram of four isolated strain PCR products. M, deoxyribonucleic acid molecular weight markers; 1, control 0 d; 2, control 31 d; 3, LAB1 31 d; 4, LAB2 31 d; 5, LAB1+LAB2 31 d.

**Figure A7:**
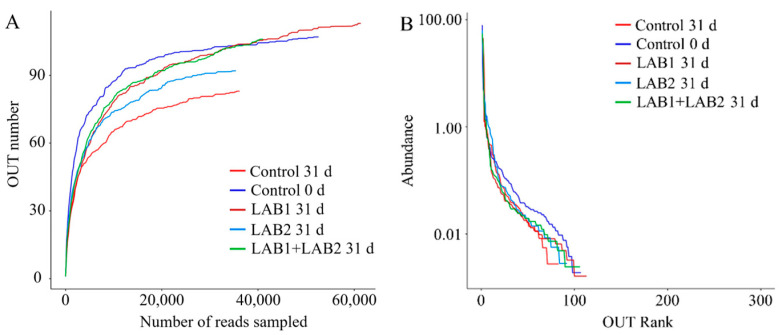
The refraction curves of operational taxonomic unit indexes (**A**) and the rank–abundance curves (**B**) of different samples.

**Figure A8:**
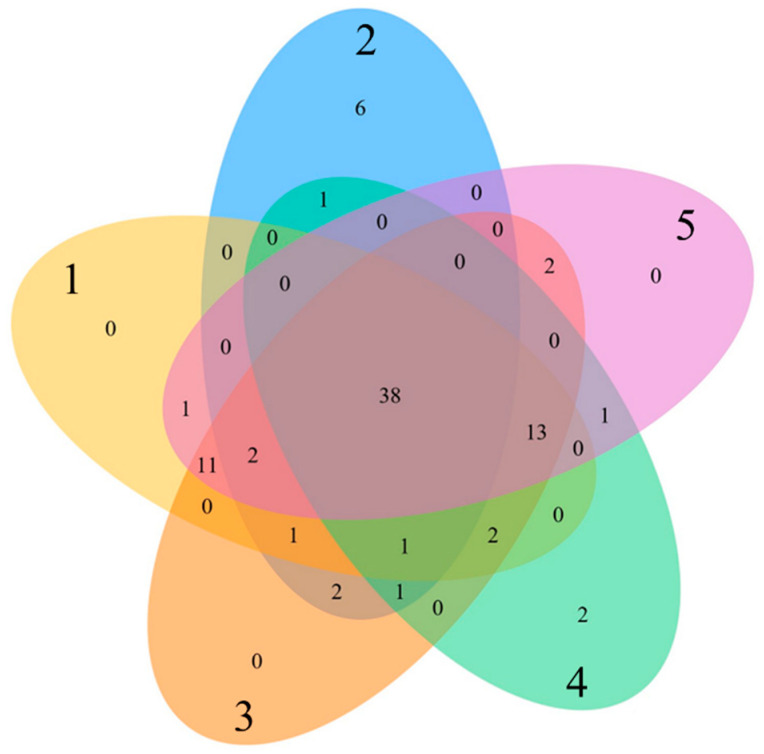
The Venn diagram for the operational taxonomic unit distribution of different samples. 1, control 0 d; 2, control 31 d; 3, LAB1 31 d; 4, LAB2 31 d; 5, LAB1+LAB2 31 d.

**Figure A9:**
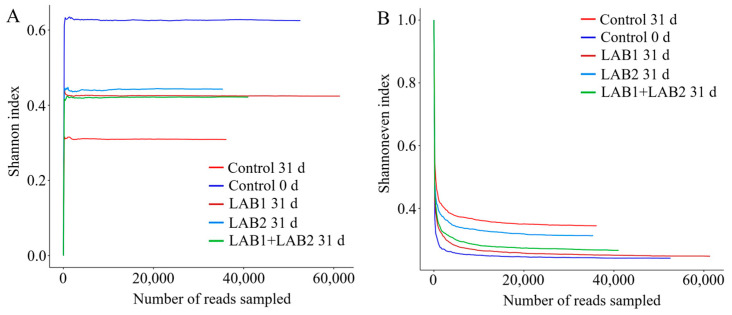
The Shannon index (**A**) and Shannoneven index (**B**) of different samples.

The DNA of five samples was extracted and the results are shown in Figure A6. Illumina MiSeq sequencing resulted in 276,192 valid sequences from five PPM samples, with an average length ranging from 406.93 to 425.86 bp. The number of OTUs with a sequence identity of 97% was consistently high across all samples, with LAB1 31 d exhibiting the highest bacterial OTU count at 113, while the control 31 d group had only 83 bacterial OTUs (Table A11). The higher OTU count in LAB1 may correlate with specific metabolic functions. The alpha diversity indices, including the Shannon index, Chao1 index, ACE index, Simpson index, and coverage, can reflect the abundance and diversity of microbial communities. As shown in Figure A7, Figure A8 and Figure A9 and Table A12, the microbial diversity indices in each sample indicated a high level of diversity, which aligned with the OTU results. Notably, the coverage rate for each sample reached 100%, confirming that the sequencing depth accurately reflected the true composition of microbial communities within all PPM samples (Table A12).

**Figure 8:**
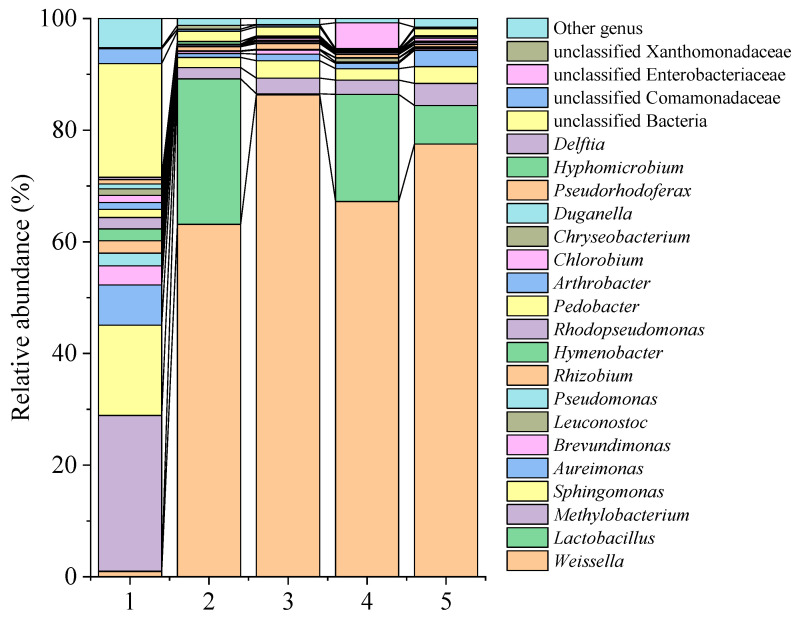
Distribution of the horizontal community structure of potherb mustard before and after fermentation. 1, control 0 d; 2, control 31 d; 3, LAB1 31 d; 4, LAB2 31 d; 5, LAB1+LAB2 31 d.

At the genus level (Figure 8), *Methylobacterium* and *Sphingomonas* were the predominant genera prior to fermentation (control 0 d), making up 27.79% and 16.16% of the sample, respectively. Following a fermentation period of 31 days, there was a significant decrease in their proportions across all groups (*p* < 0.05). In the control 31 d group, *Weissella* and *Lactobacillus* emerged as dominant genera, accounting for 65.21% and 26.05% of the sample, respectively. In LAB1 31 d, *Weissella* dominated with a proportion of 86.31%, while the proportion of *Lactobacillus* was only 0.2%, significantly lower than the other groups. In both LAB2 31 d and LAB1+LAB2 31 d, *Weissella* and *Lactobacillus* were the predominant bacterial species, accounting for 67.22% and 19.20%, and 77.52% and 6.91% of the samples, respectively (Table A13). The strong dominance of *Weissella* in LAB1 could be due to the higher acid tolerance of *Weissella* compared to *Lactobacillus*, and the inhibitory compounds produced by LAB1 that selectively favored *Weissella*. Therefore, the involvement of *Weissella* in the fermentation process of PPM is significant. It is speculated that this genus may play a major role in the fermentation of PPM.

**Figure A10:**
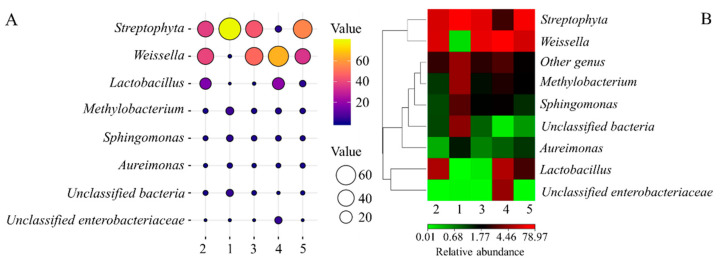

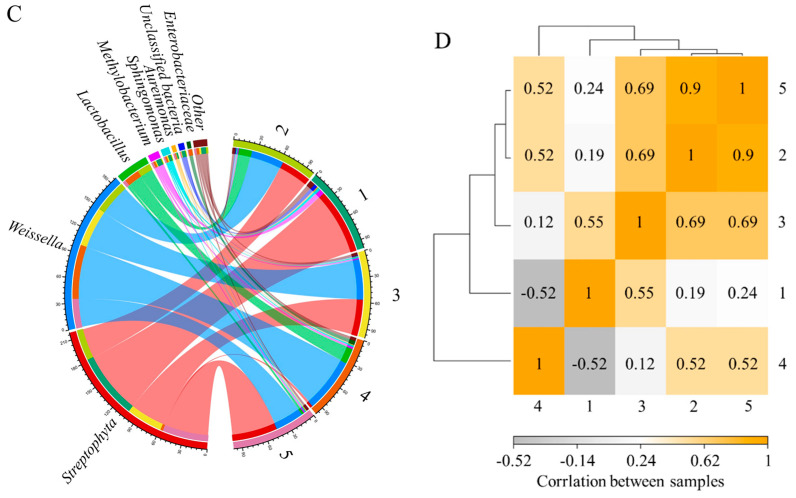
The ballon (**A**), heatmap (**B**), circos (**C**), and corrlation (**D**) of the community structure of all samples at the genus level. 1, control 0 d; 2, control 31 d; 3, LAB1 31 d; 4, LAB2 31 d; 5, LAB1+LAB2 31 d.

With regard to community structure, correlation coefficients tend to approach 1 as similarity in community structure among sample increases. The disparities in community structure between the control 0 d and LAB1+LAB2 31 d groups and between LAB1 31 d and LAB2 31 d were noteworthy. Remarkably, mixed fermentation at 31 days exhibited a striking resemblance to that of natural fermentation (Figure A10). These findings suggest that LAB2, which had limited acid-producing capability, was susceptible to contamination by undesirable bacteria, leading to a significant disparity in the composition of the microbial community. Conversely, the introduction of LAB1, which showed strong acid-producing capability, effectively controlled such contamination during fermentation, thereby promoting stabile PPM quality.

**Figure 9:**
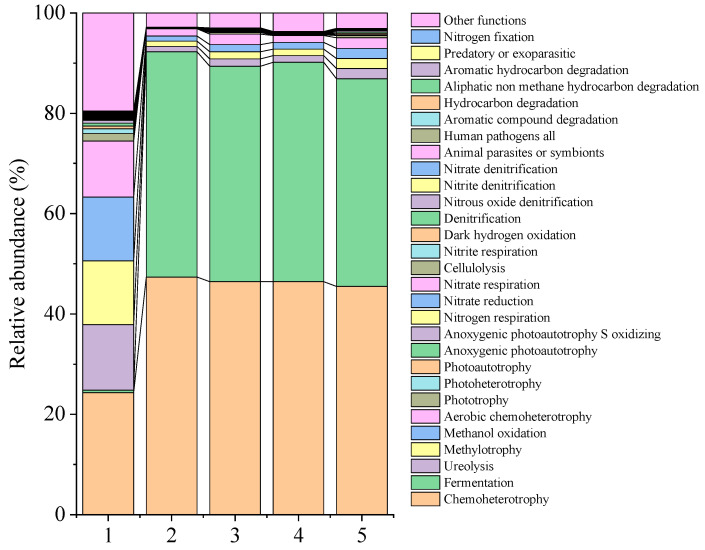
Relative abundance of microorganism functions in potherb mustard before and after fermentation. 1, control 0 d; 2, control 31 d; 3, LAB1 31 d; 4, LAB2 31 d; 5, LAB1+LAB2 31 d.

Figure 9 describes the functions of microorganisms before and after fermentation. Chemoheterotrophy and fermentation significantly increased in each group compared to control 0 d, whereas the processes of ureolysis, methylotrophy, methanol oxidation, aerobic chemoheterotrophy, and phototrophy significantly decreased (*p* < 0.05) (Table A14).

## 4. Discussion

Pickled potherb mustard is a fermented vegetable renowned for its distinctive taste. Microorganisms are the fundamental components of fermented foods, with their metabolites playing a crucial role in determining the quality [17,18]. Four strains of LAB were isolated from high-quality PPM in this study. After analyzing their fermentation characteristics, two potential starter strains were identified: the *Weissella cibaria* strain LAB1 with strong acid-producing ability and the *Leuconostoc mesenteroides* strain LAB2 with strong nitrite-degrading ability. A comparative analysis was conducted between mono-inoculated, mixed inoculated, and naturally fermented potherb mustard in regard to nutritional composition, nitrite content, microbial diversity, and other aspects. Our findings provide a theoretical foundation for industrialization and standardization of production.

A starter culture must have robust salt tolerance and acid resistance to facilitate rapid proliferation and growth during the fermentation process. *Weisseria* and *Leuconostoc mesenteroides* are widely found in fermented foods, where they play a pivotal role in the fermentation process and contribute significantly to the formation of flavor compounds [19]. *Weissella cibaria* and *Weissella confusa* are commonly found in salty fermented foods such as gray sufu and pickles, and they produce lactic acid by metabolizing glucose during fermentation, resulting in distinctive flavor profiles [20,21]. Xiang et al. [22] reported that *Weissella cibaria* and *Lactobacillus plantarum* can grow well in brine containing 6.0% salt. In our study, we isolated four strains from PPM including the *Weissella cibaria* strain LAB1 and the *Weissella confusa* strain LAB3; however, LAB1 exhibited superior tolerance to salt.

In addition, the acid-producing capacity and acid resistance of LAB1 was also the strongest among the four strains. A rapid reduction in pH results in a shorter fermentation cycle, effectively inhibiting the growth of pathogenic and spoilage bacteria in PPM. This reduces safety risks and enhances product quality. Acid tolerance is a distinct characteristic that varies significantly among strains, and the heightened tolerance of certain strains is attributable to their H^+^-ATPase activity [23].

A notable advantage of LAB is their efficient ability to hinder the proliferation of foodborne pathogens. This primarily arises from the production of a diverse range of bioactive substances, including organic acids, antimicrobial peptides, and hydrogen peroxide [24]. The antimicrobial peptides synthesized by *Weissella* exhibit antibacterial activity against both Gram-positive and Gram-negative bacteria [25,26], as confirmed in our experiments. This result was in accordance with a report by Tenea et al. [25], who found that the *W. cibaria* strain UTNGt21O produced a peptide with high capacity to inhibit the growth of *E. coli* ATCC25922 and *Salmonella enterica* ATCC51741. Also, Debasish et al. [26] demonstrated that the *W. confusa* DD_A7 strain exhibited antimicrobial activity to foodborne pathogens, including *E. coli* 0157:H7, *Listeria monocytogenes*, *Salmonella typhimurium,* and *Bacillus cereus*. Moreover, *Weisseria* demonstrates a significant inhibitory effect on fungi [24,27]. In a future work, we will comprehensively analyze the antimicrobial peptides generated by LAB1.

Both the CSs and CFSs of LAB exhibit antioxidant activity. Such activity may stem from active substances such as antioxidant enzymes or from metabolites with antioxidant activity produced during the metabolic process, such as bioactive peptides and extracellular polysaccharides. CSs tend to have higher scavenging rates of DPPH, hydroxyl radicals, and superoxide anion radicals, and greater reducing ability compared to CFSs [12,28]; our results support this notion. However, the *Weissella confusa* strain KR780676, isolated from fermented Indian food, demonstrates a superior clearance rate of hydroxyl free radicals compared to CFSs [23]. The antioxidant capacity varies significantly among different strains, which may be closely associated with their ability to secrete bioactive peptides and exopolysaccharides.

Nitrite is commonly found in various pickled vegetables, particularly leafy vegetables, and is the primary safety risk factor [29]. The formation of the “nitrite peak” during early pickle fermentation is primarily attributed to the nitrate reduction effect of certain nitrate-reducing bacteria attached to the surface of raw materials [30]. In the initial stages of fermentation, particularly natural fermentation, the pH of the system is high, resulting in the weak inhibition ability of LAB toward unwanted bacteria that possess nitrate-reducing capabilities. These active bacteria can significantly reduce a large amount of nitrate into nitrite, leading to the occurrence of the nitrite peak. Currently, it is widely accepted that LAB degrade nitrite via nitrite reductase (NiR), acid, and certain metabolites, where enzymatic degradation is the dominant process [31]. The denitrification pathway is the most frequently observed pathway for enzyme degradation in nitrite metabolism, as classified by the KEGG classification system [8,32]. Whether LAB2 also degrades nitrite through the denitrification pathway needs to be verified.

The results of community analyses based on the 16S rDNA gene revealed that *Methylobacterium* and *Sphingomonas* were the predominant microorganisms inhabiting the surface of fresh potherb mustard prior to fermentation. These microorganisms primarily originate from various environmental sources such as air, soil, and water [33]. After a fermentation period of 31 days, the predominant genera found in PPM were *Weisseria* and *Lactobacillus*, which aligns with the microbial composition commonly observed in various types of pickles [4,34]. Jung et al. [35] used a metagenomic approach to monitor the dynamic changes in the microbial community during kimchi fermentation. *Lactobacillus* and *Weisseria* were the predominant genera in the final product; they also reported that increases in the levels of lactic acid, acetic acid, ethanol, and mannitol content were associated with the metabolic activities of heterofermenting strains as they utilized free sugars. Kim et al. [36] found that *Leuconostoc mesenteroides* intestinalis dominated during the initial phase of kimchi fermentation while *Lactobacillus* exhibited more robust growth in the later stages of fermentation. Park et al. [37] reported that *Leuconostoc mesenteroides*, *Lactobacillus plantarum*, and *Lactobacillus brevis* were predominant strains on days 1, 5, and 10 of kimchi fermentation, respectively. In summary, *Leuconostoc mesenteroides*, *Weissella*, and *Lactobacillus* are usually the dominant microorganisms in kimchi, which play their respective roles in different stages and cooperate to complete the fermentation process.

The nitrogen content of amino acids is closely associated with the flavor of PPM, primarily derived from aromatic compounds in raw materials and inorganic salts, as well as amino acids, organic acids, and sugars produced during fermentation [38]. In addition, amino acids can serve as precursors for reacting with other compounds, thereby generating additional flavor substances that influence taste. According to Liu et al. [3], *Weissella* and *Lactobacillus* abundances were positively related to metabolites important in fermented potherb mustard flavoring, such as amino acids, organic acids, etc. In our study, groups inoculated with LAB1 as a starter culture exhibited significantly elevated levels of NAAs compared to the naturally fermented group. This finding further substantiates the positive impact of LAB1 on potherb mustard fermentation and its capacity to enhance quality. Further research is needed, however, to dissect the precise regulating mechanism of LAB inoculation fermentation in the accumulation of important metabolites in potherb mustard, which might extend our knowledge of the quality and flavor formation of traditional fermented vegetables.

## 5. Conclusions

The *Weissella cibaria* strain LAB1 exhibited the most robust acid-producing ability, antibacterial capacity, and antioxidant activity, and the *Leuconostoc mesenteroides* strain LAB2 demonstrated the highest nitrite-degrading ability. Inoculation of either LAB1 alone or in combination with LAB2 effectively mitigated bacterial contamination during fermentation. This indicates that LAB1 is an exceptional starter culture that leads to superior fermentation. Consequently, it is highly suitable for use as a mono-starter for PPM. The LAB2 strain had a strong ability to degrade nitrite but had poor acid-producing ability. The products obtained by mono-inoculated LAB2 did not show suitable microbial community structure or NAA levels for use as a starter culture. However, it showed a combined effect with LAB1, promoting the proliferation of *Lactobacillus* and *Weissella* in the final product, while significantly reducing nitrite levels. Thus, LAB2 is an effective synergistic starter culture for the production of PPM. In conclusion, the inoculation of LAB1 and LAB2 improved the quality and safety of pickled potherb mustard by promoting the growth of LAB, stabilizing bacterial community, increasing TA and NAA contents, shortening fermentation time, and reducing nitrite content. The results provide a theoretical foundation for the commercial application of these starter cultures.

## Figures and Tables

**Table 1:** Homologous alignment analysis of 16S rRNA sequences of four lactic acid bacteria strains isolated from the pickled potherb mustard.

Strain	Accession	Strain	Sequence Length/bp	Query Cover/%	Percentage of Identification/%
LAB1	MN700179.1	*Weissella cibaria* strain UPMC1238	1413	100	99.93
LAB2	KU361186.1	*Leuconostoc mesenteroides* strain TA	1510	100	100
LAB3	MT613505.1	*Weissella cibaria* strain 3128	855	100	100
LAB4	MT545113.1	*Leuconostoc mesenteroides* strain 4622	1391	100	100

**Table 2:** Inhibition of lactic acid bacteria on *Escherichia coli* and *Staphylococcus aureus* strains.

Strain	Antibacterial Circle Diameters/mm	
	*E. coli* CCTCC AB 2014080	*S. aureus* CCTCC AB 2010020
LAB1	14.03 ± 0.58 a	12.55 ± 0.82 a
LAB2	12.83 ± 0.74 c	12.32 ± 0.39 b
LAB3	13.40 ± 0.81 b	12.46 ± 0.86 a
LAB4	11.13 ± 0.63 d	11.97 ± 0.29 c

Note: Data are expressed as means of triplicate samples ± SD. Mean values with different lowercase letters in the same column are significantly different (*p* < 0.05).

**Table 3:** The peptides in fermentation broth with a Peptide Ranker score of more than 0.5 after fermentation.

No.	Protein Accession	Peptides	Peptide Ranker (Bioactive)	Length	Mass	Percentage of HAAs (%)
1	tr|A0A1X4JKK3|A0A1X4JKK3_9LACO	S.PTFPDPL.G	0.865	7	785.3959	71.43
2	tr|A0A2S1KSC4|A0A2S1KSC4_9LACO	N.PGLVPPL.S	0.794	7	691.4268	85.71
3	tr|A0A0N9Y818|A0A0N9Y818_9LACO	P.KDPSQGYWPPT.V	0.739	11	1274.5931	45.45
4	tr|A0A0D1LI50|A0A0D1LI50_9LACO	V.GPAVIAMVM.A	0.701	9	887.4609	88.89
5	tr|A0A1X4JKK3|A0A1X4JKK3_9LACO	E.PGSPVLP.H	0.682	7	665.3748	71.43
6	tr|A0A0D1LZ28|A0A0D1LZ28_9LACO	G.EGWTLWNGNPIP.S	0.674	12	1382.6619	50
7	tr|A0A0N9Y5P2|A0A0N9Y5P2_9LACO	L.TFWLDGK.A	0.604	7	865.4333	42.86
8	tr|A0A0D1K432|A0A0D1K432_9LACO	G.KDPVAKPGDGGYWAT.Y	0.59	15	1560.7572	40
9	tr|A0A2S1KTI5|A0A2S1KTI5_9LACO	N.GPATDIPAG.Y	0.565	9	797.3919	55.56
10	tr|A0A2S1KTI5|A0A2S1KTI5_9LACO	N.LNGPYGPK.V	0.54	8	844.4443	37.5
11	tr|A0A1X4JKK3|A0A1X4JKK3_9LACO	Y.TPEGPNGPL.T	0.512	9	880.429	44.44
12	tr|A0A2S1KNI5|A0A2S1KNI5_9LACO	L.VGDVGFG.K	0.501	7	649.3071	42.86

Note: HAAs: hydrophobic amino acids.

## Data Availability

The original contributions presented in this study are included in the article; further inquiries can be directed to the corresponding author/s.

## Appendix A

**Table A1:** Design factors and coded levels of LAB1 for response surface experiments.

Independent Variables	Design Factors	Ranges and Levels		
		Low Value (−1)	0	High Value (+1)
Temperature (°C)	A	19.0	28.0	37.0
Inoculation amount (%)	B	2.0	3.0	4.0
Initial pH	C	4.0	5.0	6.0

**Table A2:** The results of total acid analyses (LAB1).

Run	Variables			Responses
	Temperature (A)	Inoculation Amount (B)	Initial pH (C)	Total Acid (g L^−1^)
1	−1	−1	0	50.63
2	1	−1	0	78.75
3	−1	1	0	47.25
4	1	1	0	83.25
5	−1	0	−1	70.88
6	1	0	−1	74.25
7	−1	0	1	34.88
8	1	0	1	76.50
9	0	−1	−1	72.00
10	0	1	−1	74.25
11	0	−1	1	81.00
12	0	1	1	76.50
13	0	0	0	97.88
14	0	0	0	95.63
15	0	0	0	87.75
16	0	0	0	90.00
17	0	0	0	91.13

Note: Y = 9.25 + 1.36A − 0.0141B − 0.2813C + 0.1969AB + 0.9563AC − 0.1687BC − 1.97A2 − 0.7847B2 − 0.8691C2. The optimal culture conditions were temperature 31.21 °C, inoculation amount 3.03%, initial pH 5.03, and TA content 94.86 g L^−1^.

**Table A3:** Analysis of variance (ANOVA) for regression equations (LAB1).

Source	Sum of Squares	DF	Mean Square	F Value	*p*-Value	Significant
Model	43.47	9	4.83	9.15	0.0040	**
A	14.89	1	14.89	28.19	0.0011	**
B	0.0016	1	0.0016	0.0030	0.9579	
C	0.6328	1	0.6328	1.20	0.3099	
AB	0.1550	1	0.1550	0.2936	0.6047	
AC	3.66	1	3.66	6.93	0.0338	*
BC	0.1139	1	0.1139	0.2157	0.6564	
A^2^	16.27	1	16.27	30.82	0.0009	**
B^2^	2.59	1	2.59	4.91	0.0623	
C^2^	3.18	1	3.18	6.02	0.0438	*
Residual	3.70	7	0.5280			
Lack of Fit	3.00	3	1.00	5.77	0.0617	
Pure Error	0.6936	4	0.1734			
Cor Total	47.17	16				

Note: * and ** indicate significant correlation at the 0.05 and 0.01 level, respectively.

**Table A4:** Design factors and coded levels of LAB2 for response surface experiments.

Independent Variables	Design Factors	Ranges and Levels		
		Low Value (−1)	0	High Value (+1)
Temperature (°C)	A	19.0	28.0	37.0
Inoculation amount (%)	B	4.0	5.0	6.0
Initial pH	C	5.0	6.0	7.0

**Table A5:** The results of total acid analyses (LAB2).

Run	Variables			Responses
	Temperature (A)	Inoculation Amount (B)	Initial pH (C)	Total Acid (g L^−1^)
1	−1	−1	0	68.63
2	1	−1	0	81.00
3	−1	1	0	63.00
4	1	1	0	76.50
5	−1	0	−1	55.13
6	1	0	−1	59.63
7	−1	0	1	68.63
8	1	0	1	75.38
9	0	−1	−1	79.88
10	0	1	−1	77.63
11	0	−1	1	77.63
12	0	1	1	82.13
13	0	0	0	88.88
14	0	0	0	93.38
15	0	0	0	90.00
16	0	0	0	90.00
17	0	0	0	92.25

Note: Y = 9.09 + 0.4641A − 0.0984B + 0.3937C + 0.0281AB + 0.0563AC + 0.1688BC − 1.66A2 − 0.1997B2 − 0.9591C2. The optimal culture conditions were temperature 29.28 °C, inoculation amount 4.85%, initial pH 6.20, and TA content 91.62 g L^−1^.

**Table A6:** Analysis of variance (ANOVA) for regression equations (LAB2).

Source	Sum of Squares	DF	Mean Square	F Value	*p*-Value	Significant
Model	19.93	9	2.21	10.29	0.0028	**
A	1.72	1	1.72	8.00	0.0254	**
B	0.0775	1	0.0775	0.3601	0.5673	
C	1.24	1	1.24	5.76	0.0474	
AB	0.0032	1	0.0032	0.0147	0.9069	
AC	0.0127	1	0.0127	0.0588	0.8154	*
BC	0.1139	1	0.1139	0.5292	0.4906	
A^2^	11.63	1	11.63	54.05	0.0002	**
B^2^	0.1679	1	0.1679	0.7800	0.4064	
C^2^	3.87	1	3.87	17.99	0.0038	*
Residual	1.51	7	0.2152			
Lack of Fit	1.37	3	0.4567	13.36	0.0150	
Pure Error	0.1367	4	0.0342			
Cor Total	21.44	16				

Note: * and ** indicate significant correlation at the 0.05 and 0.01 level, respectively.

**Table A7:** Characteristics of colony morphology.

Strain	Diameter/mm	Appearance	Color	Edge	Adhesion
LAB1	0.76 ± 0.04	Circular, moist, and glossy	Milk white	Neat and regular	Weak
LAB2	0.35 ± 0.06	Circular, smooth, and moist	White	Neat and regular	Weak
LAB3	1.40 ± 0.08	Circular, moist, and glossy	Milk white	Neat and regular	Weak
LAB4	0.17 ± 0.02	Circular, smooth, and moist	White	Neat and regular	Weak

**Table A8:** Biochemical and physiological characteristics of the lactic acid bacteria isolated from pickled potherb mustard.

Tests	Characteristics			
	LAB1	LAB2	LAB3	LAB4
Catalase activity	−	−	−	−
Growth in 65 g L^−1^ NaCl	+	+	+	+
Bacterial motility test	−	−	−	−
Nitrate reduction	−	−	−	−
Acid and gas production by fermentation of glucose	+	+	+	+
Hydrogen sulfide production	−	−	−	−
Gelatin liquefaction	−	−	−	−
Indole test	−	−	−	−
Bile esculin test	+	+	+	+

Note: +, positive reaction; −, negative reaction.

**Table A9:** Nitrite degradation ability of the lactic acid bacteria isolated from pickled potherb mustard.

Culture Duration/h	Nitrite Degradation Rate/%			
	LAB1	LAB2	LAB3	LAB4
12	26.43 ± 4.57 b	89.63 ± 4.63 a	8.95 ± 2.58 d	21.53 ± 3.11 c
24	73.55 ± 2.53 b	90.46 ± 5.31 a	38.17 ± 3.81 d	63.06 ± 6.76 c
36	87.11 ± 4.34 b	91.16 ± 5.35 a	61.94 ± 8.67 d	77.32 ± 4.17 c
48	91.02 ± 2.75 a	91.72 ± 6.87 a	79.7 ± 3.14 c	86.83 ± 6.32 b
60	91.58 ± 4.27 a	91.86 ± 4.36 a	87.39 ± 6.08 c	89.9 ± 5.34 b
72	91.72 ± 3.65 b	92 ± 2.72 a	90.32 ± 6.18 c	90.32 ± 4.58 c

Note: Data are expressed as means ± SD of triplicate assays. Mean values with different lowercase letters in same row are significantly different (*p* < 0.05).

**Table A10:** The in vitro antioxidant capacities of the lactic acid bacteria isolated from pickled potherb mustard.

Free Radical Scavenging Rate/%		Strains			
		LAB1	LAB2	LAB3	LAB4
DPPH	CSs	33.07 ± 5.91 a	25.68 ± 1.85 b	11.67 ± 2.56 d	15.18 ± 2.20 c
	CFE	19.07 ± 3.34 a	15.95 ± 4.85 b	4.67 ± 3.73 d	8.56 ± 1.95 c
OH	CSs	22.26 ± 3.35 a	14.46 ± 2.87 c	16.10 ± 2.03 b	16.70 ± 2.91 b
	CFE	21.61 ± 4.94 a	13.96 ± 2.84 b	11.41 ± 3.46 c	13.29 ± 1.65 b
O_2_^·−^	CSs	52.21 ± 5.53 a	21.59 ± 7.27 d	37.52 ± 5.89 b	27.62 ± 3.00 c
	CFE	13.81 ± 4.27 c	17.00 ± 6.00 a	9.38 ± 1.69 d	14.87 ± 2.88 b
Reducing ability	CSs	54.29 ± 4.84 a	40.00 ± 5.45 b	20.00 ± 2.67 d	22.86 ± 2.26 c
	CFE	11.43 ± 2.13 b	20.00 ± 2.46 a	2.86 ± 1.94 d	5.71 ± 2.50 c

Data are expressed as means ± SD of triplicate assays. Mean values with different lowercase letters in same row are significantly different (*p* < 0.05).

**Table A11:** Statistics of effective sequence data of microorganisms from potherb mustard before and after fermentation.

Samples	Barcode	Sequence Number	Base Number	Mean Length	Min Length	Max Length	OUTs
Control 0 d	AGAACA	64,812	26,373,965	406.93	356	469	107.0
Control 31 d	TATCGA	46,099	19,313,956	418.97	350	446	83.0
LAB1 31 d	GGTGTG	72,345	30,161,842	416.92	352	446	113.0
LAB2 31 d	AGAGAC	45,003	19,164,849	425.86	352	451	92.0
LAB1+LAB2 31 d	TATGCA	47,933	19,888,245	414.92	350	453	106.0

**Table A12:** The alpha diversity indices for sequencing of potherb mustard before and after fermentation.

Samples	Number	Shannon	Chao1	Ace	Simpson	Shannoneven	Coverage
Control 0 d	52,578.0	1.13	116.0	112.39	0.63	0.24	1.00
Control 31 d	36,163.0	1.52	96.0	91.77	0.31	0.34	1.00
LAB1 31 d	61,354.0	1.17	123.11	119.45	0.42	0.25	1.00
LAB2 31 d	35,390.0	1.42	95.6	96.14	0.44	0.31	1.00
LAB1+LAB2 31 d	41,042.0	1.24	121.11	115.92	0.42	0.27	1.00

**Table A13:** Relative abundance (%) of bacterial in potherb mustard before and after fermentation.

Genus	CK 0 d	CK 31 d	LAB1 31 d	LAB2 31 d	LAB1+LAB2 31 d
*Weissella*	0.94	63.14	86.31	67.22	77.52
*Lactobacillus*	0.05	26.05	0.20	19.20	6.91
*Methylobacterium*	27.92	2.02	2.81	2.53	3.95
*Sphingomonas*	16.16	1.82	3.09	2.06	3.02
*Aureimonas*	7.22	0.72	1.21	0.98	2.92
*Brevundimonas*	3.38	0.37	0.72	0.21	0.27
*Leuconostoc*	0.01	0.06	0.01	0.74	0.13
*Pseudomonas*	2.30	0.03	0.15	0.04	0.21
*Rhizobium*	2.21	0.75	1.04	0.61	0.41
*Hymenobacter*	2.12	0.01	0.08	0.09	0.05
*Rhodopseudomonas*	2.06	0.01	0.32	0.24	0.33
*Pedobacter*	1.43	0.08	0.16	0.10	0.19
*Arthrobacter*	1.27	0.27	0.03	0.05	0.04
*Chlorobium*	1.27	0.00	0.35	0.06	0.56
*Chryseobacterium*	1.19	0.06	0.09	0.03	0.26
*Duganella*	0.86	0.03	0.13	0.04	0.07
*Pseudorhodoferax*	0.77	0.00	0.01	0.01	0.01
*Hyphomicrobium*	0.00	0.48	0.00	0.00	0.00
*Delftia*	0.42	0.00	0.13	0.05	0.10
*unclassified Bacteria*	20.37	1.83	1.65	0.12	1.23
*unclassified Comamonadaceae*	2.63	0.35	0.38	0.22	0.25
*unclassified Enterobacteriaceae*	0.20	0.03	0.04	4.60	0.04
*unclassified Xanthomonadaceae*	0.04	0.66	0.01	0.00	0.01
*Other genus*	5.18	1.25	1.07	0.79	1.55

**Table A14:** Relative abundance (%) of microorganism functions in potherb mustard before and after fermentation.

Functions	CK 0d	CK 31 d	LAB1 31 d	LAB2 31 d	LAB1+LAB2 31 d
Chemoheterotrophy	24.32	47.35	46.44	46.42	45.50
Fermentation	0.51	44.92	42.94	43.77	41.39
Ureolysis	13.04	1.05	1.47	1.30	2.04
Methylotrophy	12.72	1.05	1.42	1.31	2.01
Methanol oxidation	12.72	1.05	1.42	1.31	2.01
Aerobic chemoheterotrophy	11.16	1.39	2.08	1.38	2.13
Phototrophy	1.50	0.01	0.33	0.15	0.43
Photoheterotrophy	0.91	0.01	0.15	0.12	0.16
Photoautotrophy	0.56	0.00	0.17	0.03	0.27
Anoxygenic photoautotrophy	0.56	0.00	0.17	0.03	0.27
Anoxygenic photoautotrophy S oxidizing	0.56	0.00	0.17	0.03	0.27
Nitrogen respiration	0.16	0.03	0.03	0.05	0.05
Nitrate reduction	0.16	0.03	0.03	0.05	0.05
Nitrate respiration	0.16	0.03	0.03	0.05	0.05
Cellulolysis	0.13	0.01	0.01	0.04	0.03
Nitrite respiration	0.13	0.01	0.01	0.04	0.03
Dark hydrogen oxidation	0.13	0.01	0.01	0.04	0.03
Denitrification	0.13	0.01	0.01	0.04	0.03
Nitrous oxide denitrification	0.13	0.01	0.01	0.04	0.03
Nitrite denitrification	0.13	0.01	0.01	0.04	0.03
Nitrate denitrification	0.13	0.01	0.03	0.00	0.02
Animal parasites or symbionts	0.11	0.00	0.01	0.02	0.03
Human pathogens (all)	0.11	0.00	0.01	0.02	0.03
Aromatic compound degradation	0.08	0.03	0.01	0.01	0.02
Hydrocarbon degradation	0.05	0.01	0.01	0.00	0.00
Aliphatic non-methane hydrocarbon degradation	0.05	0.01	0.01	0.00	0.00
Aromatic hydrocarbon degradation	0.05	0.01	0.01	0.00	0.00
Predatory or exoparasitic	0.00	0.05	0.00	0.00	0.00
Nitrogen fixation	0.00	0.00	0.00	0.00	0.03
Other functions	19.54	2.86	2.97	3.70	3.05

## Appendix B

- Deoxyribonucleic acid sequence of LAB1

GGAAACCTACCTCTTAGCAGGGGGATAACATTTGGAAACAGATGCTAATACCGTATAACAATAGCAACCGCATGGTTGCTACTTAAAAGATGGTTCTGCTATCACTAAGAGATGGTCCCGCGGTGCATTAGTTAGTTGGTGAGGTAATGGCTCACCAAGACGATGATGCATAGCCGAGTTGAGAGACTGATCGGCCACAATGGGACTGAGACACGGCCCATACTCCTACGGGAGGCAGCAGTAGGGAATCTTCCACAATGGGCGAAAGCCTGATGGAGCAACGCCGCGTGTGTGATGAAGGGTTTCGGCTCGTAAAACACTGTTGTAAGAGAAGAATGACATTGAGAGTAACTGTTCAATGTGTGACGGTATCTTACCAGAAAGGAACGGCTAAATACGTGCCAGCAGCCGCGGTAATACGTATGTTCCAAGCGTTATCCGGATTTATTGGGCGTAAAGCGAGCGCAGACGGTTATTTAAGTCTGAAGTGAAAGCCCTCAGCTCAACTGAGGAATTGCTTTGGAAACTGGATGACTTGAGTGCAGTAGAGGAAAGTGGAACTCCATGTGTAGCGGTGAAATGCGTAGATATATGGAAGAACACCAGTGGCGAAGGCGGCTTTCTGGACTGTAACTGACGTTGAGGCTCGAAAGTGTGGGTAGCAAACAGGATTAGATACCCTGGTAGTCCACACCGTAAACGATGAGTGCTAGGTGTTTGAGGGTTTCCGCCCTTAAGTGCCGCAGCTAACGCATTAAGCACTCCGCCTGGGGAGTACGACCGCAAGGTTGAAACTCAAAGGAATTGACGGGGACCCGCACAAGCGGTGGAGCATGTGGTTTAATTCGAAGCAACGCGAAGAACCTTACCAGGTCTTGACATCCCTTGACAACTCCAGAGATGGAGCGTTCCCTTCGGGGACAAGGTGACAGGTGGTGCATGGTTGTCGTCAGCTCGTGTCGTGAGATGTTGGGTTAAGTCCCGCAACGAGCGCAACCCTTATTACTAGTTGCCAGCATTTAGTTGGGCACTCTAGTGAGACTGCCGGTGACAAACCGGAGGAAGGTGGGGATGACGTCAAATCATCATGCCCCTTATGACCTGGGCTACACACGTGCTACAATGGCGTATACAACGAGTTGCCAACCCGCGAGGGTGAGCTAATCTCTTAAAGTACGTCTCAGTTCGGATTGTAGGCTGCAACTCGCCTACATGAAGTCGGAATCGCTAGTAATCGCGGATCAGCACGCCGCGGTGAATACGTTCCCGGGTCTTGTACACACCGCCCGTCACACCATGAGAGTTTGTAACACCCAAAGCCGGTGGGGTAACCTTCGGGAGCCAGCCGTCTAAGGTGGGACAGATGATTAGGGTGAAGTCGTAACAAGGTAGCCGTAGGAGAACCTGCGGCTG

- Deoxyribonucleic acid sequence of LAB2

GCTCAGGATGAACGCTGGCGGCGTGCCTAATACATGCAAGTCGAACGCACAGCGAAAGGTGCTTGCACCTTTCAAGTGAGTGGCGAACGGGTGAGTAACACGTGGACAACCTGCCTCAAGGCTGGGGATAACATTTGGAAACAGATGCTAATACCGAATAAAACTTAGTGTCGCATGACACAAAGTTAAAAGGCGCTTCGGCGTCACCTAGAGATGGATCCGCGGTGCATTAGTTAGTTGGTGGGGTAAAGGCCTACCAAGACAATGATGCATAGCCGAGTTGAGAGACTGATCGGCCACATTGGGACTGAGACACGGCCCAAACTCCTACGGGAGGCTGCAGTAGGGAATCTTCCACAATGGGCGAAAGCCTGATGGAGCAACGCCGCGTGTGTGATGAAGGCTTTCGGGTCGTAAAGCACTGTTGTATGGGAAGAACAGCTAGAATAGGAAATGATTTTAGTTTGACGGTACCATACCAGAAAGGGACGGCTAAATACGTGCCAGCAGCCGCGGTAATACGTATGTCCCGAGCGTTATCCGGATTTATTGGGCGTAAAGCGAGCGCAGACGGTTTATTAAGTCTGATGTGAAAGCCCGGAGCTCAACTCCGGAATGGCATTGGAAACTGGTTAACTTGAGTGCAGTAGAGGTAAGTGGAACTCCATGTGTAGCGGTGGAATGCGTAGATATATGGAAGAACACCAGTGGCGAAGGCGGCTTACTGGACTGCAACTGACGTTGAGGCTCGAAAGTGTGGGTAGCAAACAGGATTAGATACCCTGGTAGTCCACACCGTAAACGATGAACACTAGGTGTTAGGAGGTTTCCGCCTCTTAGTGCCGAAGCTAACGCATTAAGTGTTCCGCCTGGGGAGTACGACCGCAAGGTTGAAACTCAAAGGAATTGACGGGGACCCGCACAAGCGGTGGAGCATGTGGTTTAATTCGAAGCAACGCGAAGAACCTTACCAGGTCTTGACATCCTTTGAAGCTTTTAGAGATAGAAGTGTTCTCTTCGGAGACAAAGTGACAGGTGGTGCATGGTCGTCGTCAGCTCGTGTCGTGAGATGTTGGGTTAAGTCCCGCAACGAGCGCAACCCTTATTGTTAGTTGCCAGCATTCAGATGGGCACTCTAGCGAGACTGCCGGTGACAAACCGGAGGAAGGCGGGGACGACGTCAGATCATCATGCCCCTTATGACCTGGGCTACACACGTGCTACAATGGCGTATACAACGAGTTGCCAACCCGCGAGGGTGAGCTAATCTCTTAAAGTACGTCTCAGTTCGGATTGTAGTCTGCAACTCGACTACATGAAGTCGGAATCGCTAGTAATCGCGGATCAGCACGCCGCGGTGAATACGTTCCCGGGTCTTGTACACACCGCCCGTCACACCATGGGAGTTTGTAATGCCCAAAGCCGGTGGCCTAACCTTTTAGGAAGGAGCCGTCTAAGGCAGGACAGATGACTGGGGTGAAGTCGTAACAAGGTAGCCGTAGGAGAACCT

- Deoxyribonucleic acid sequence of LAB3

CGCTTTGTGGTTCAACTGATTTGAAGAGCTTGCTCAGATATGACGATGGACATTGCAAAGAGTGGCGAACGGGTGAGTAACACGTGGGAAACCTACCTCTTAGCAGGGGATAACATTTGGAAACAGATGCTAATACCGTATAACAATAGCAACCGCATGGTTGCTACTTAAAAGATGGTTCTGCTATCACTAAGAGATGGTCCCGCGGTGCATTAGTTAGTTGGTGAGGTAATGGCTCACCAAGACGATGATGCATAGCCGAGTTGAGAGACTGATCGGCCACAATGGGACTGAGACACGGCCCATACTCCTACGGGAGGCAGCAGTAGGGAATCTTCCACAATGGGCGAAAGCCTGATGGAGCAACGCCGCGTGTGTGATGAAGGGTTTCGGCTCGTAAAACACTGTTGTAAGAGAAGAATGACATTGAGAGTAACTGTTCAATGTGTGACGGTATCTTACCAGAAAGGAACGGCTAAATACGTGCCAGCAGCCGCGGTAATACGTATGTTCCAAGCGTTATCCGGATTTATTGGGCGTAAAGCGAGCGCAGACGGTTATTTAAGTCTGAAGTGAAAGCCCTCAGCTCAACTGAGGAATTGCTTTGGAAACTGGATGACTTGAGTGCAGTAGAGGAAAGTGGAACTCCATGTGTAGCGGTGAAATGCGTAGATATATGGAAGAACACCAGTGGCGAAGGCGGCTTTCTGGACTGTAACTGACGTTGAGGCTCGAAAGTGTGGGTAGCAAACAGGATTAGATACCCTGGTAGTCCACACCGTAAACGATGAGTGCTAGGTGTTTGAGGGTTTCCGCCCTTAAGTGCCGCAGCTAACGCATTAAGCACTCCGCCTG

- Deoxyribonucleic acid sequence of LAB4

CGCACAGCGAAAGGTGCTTGCACCTTTCAAGTGAGTGGCGAACGGGTGAGTAACACGTGGACAACCTGCCTCAAGGCTGGGGATAACATTTGGAAACAGATGCTAATACCGAATAAAACTTAGTGTCGCATGACACAAAGTTAAAAGGCGCTTCGGCGTCACCTAGAGATGGATCCGCGGTGCATTAGTTAGTTGGTGGGGTAAAGGCCTACCAAGACAATGATGCATAGCCGAGTTGAGAGACTGATCGGCCACATTGGGACTGAGACACGGCCCAAACTCCTACGGGAGGCTGCAGTAGGGAATCTTCCACAATGGGCGAAAGCCTGATGGAGCAACGCCGCGTGTGTGATGAAGGCTTTCGGGTCGTAAAGCACTGTTGTATGGGAAGAACAGCTAGAATAGGAAATGATTTTAGTTTGACGGTACCATACCAGAAAGGGACGGCTAAATACGTGCCAGCAGCCGCGGTAATACGTATGTCCCGAGCGTTATCCGGATTTATTGGGCGTAAAGCGAGCGCAGACGGTTTATTAAGTCTGATGTGAAAGCCCGGAGCTCAACTCCGGAATGGCATTGGAAACTGGTTAACTTGAGTGCAGTAGAGGTAAGTGGAACTCCATGTGTAGCGGTGGAATGCGTAGATATATGGAAGAACACCAGTGGCGAAGGCGGCTTACTGGACTGCAACTGACGTTGAGGCTCGAAAGTGTGGGTAGCAAACAGGATTAGATACCCTGGTAGTCCACACCGTAAACGATGAACACTAGGTGTTAGGAGGTTTCCGCCTCTTAGTGCCGAAGCTAACGCATTAAGTGTTCCGCCTGGGGAGTACGACCGCAAGGTTGAAACTCAAAGGAATTGACGGGGACCCGCACAAGCGGTGGAGCATGTGGTTTAATTCGAAGCAACGCGAAGAACCTTACCAGGTCTTGACATCCTTTGAAGCTTTTAGAGATAGAAGTGTTCTCTTCGGAGACAAAGTGACAGGTGGTGCATGGTCGTCGTCAGCTCGTGTCGTGAGATGTTGGGTTAAGTCCCGCAACGAGCGCAACCCTTATTGTTAGTTGCCAGCATTCAGATGGGCACTCTAGCGAGACTGCCGGTGACAAACCGGAGGAAGGCGGGGACGACGTCAGATCATCATGCCCCTTATGACCTGGGCTACACACGTGCTACAATGGCGTATACAACGAGTTGCCAACCCGCGAGGGTGAGCTAATCTCTTAAAGTACGTCTCAGTTCGGATTGTAGTCTGCAACTCGACTACATGAAGTCGGAATCGCTAGTAATCGCGGATCAGCACGCCGCGGTGAATACGTTCCCGGGTCTTGTACACACCGCCCGTCACACCATGGGAGTTTGTAATGCCCAAAGCCGGTGGCCTAACC
